# Supplementary material for: Soluble Epoxide Hydrolase in Aged Female Mice and Human Explanted Hearts Following Ischemic Injury
Source: Int J Mol Sci. 2021 Feb 8;22(4):1691. doi: 10.3390/ijms22041691 (PMC7915306; doi:10.3390/ijms22041691)
Supplement: Supplementary file 1 [file ijms-22-01691-s001.pdf]

Supplemental Data

Supplemental Table S1. Clinical parameters for non-failing control (NFC) and ischemic cardiomyopathy (ICM) human transplant hearts.

**NFC**		
Variable	Female (n=5)	IQR; %
Demographic		
Age at transplant	48.0	31.5-59.5
Anthropometric		
Weight (kg)	67.2	64.0-69.5
Height (m)	1.62	1.59-1.71
BMI (kg/m^2)	24.4	23.8-26.6
**ICM**		
Variable	Females (n=5)	IQR; %
Demographic		
Age at transplant	58.0	54.0-61.0
Anthropometric		
Weight (kg)	73.9	53.5-78.5
Height (m)	162.0	160.0-164.5
BMI (kg/m^2)	26.5	20.6-30.3
Physical assessment		
HR (bpm)	90.0	79.5-110.5
BP (syst)	127.5	110.8-148.0
BP (diast)	62.5	48.3-79.5
NYHA class III (%)	1	20
NYHA class IV (%)	4	80
Comorbidities (%)		
COPD/Asthma	0	0
DM	1	20
Dyslipidemia	2	40
Kidney disease	3	60
HTN	0	0
Liver Disease	1	20
Echocardiography		
EF (%)	24.4	15.0-42.0
LVID;d (mm)	51.0	42.0-52.0
LVID;s (mm)	39.0	32.0-46.0
Devices (%)		
Pacemaker/ICD	2	40
ICD	1	20
BiV-ICD	0	0
Medications		
ACEI/ARB (%)	2	40
Beta Blocker (%)	4	80
eGFR (ml/min)	41.0	36.0-64.0
Creatinine (μmol/L)	121.0	88.5-138.0

Supplemental Table 2. Oxylipid metabolite profile (ng/g tissue) in human and mouse left ventricular tissue measured by LC-MS/MS. Data are shown as mean ± SEM, N = 3-6. For female human data, data is from one-way ANOVA with Tukey's post-hoc test. For mouse data, data is from two-way ANOVA with Tukey's post-hoc test. In all cases, $P \leq 0.05$, *vs control; # vs WT group; ‡ vs non-infarct; † vs peri-infarct group.

	Human				***Mouse***			
					**WT**		**sEH null**	
	NFC	Non-Infarct	Peri-Infarct	Infarct	Control	Non-Infarct	Control	Non-Infarct
Arachidonic Acid	$2.66{\times}10^6 \pm 0.38{\times}10^6$	$1.07{\times}10^6 \pm 0.24{\times}10^6$ *	$1.45{\times}10^6 \pm 0.44{\times}10^6$	$0.65{\times}10^6 \pm 0.16{\times}10^6$ *	1326.89 ± 305.87	859.63 ± 221.34	1402.92 ± 379.11	1286.38 ± 279.61
Epoxygenase-dependent metabolism								
12,13-EpOME	19.12 ± 3.15	142.70 ± 30.11*	68.43 ± 42.08	54.61 ± 15.01	5.09 ± 0.79	3.53 ± 0.60	12.64 ± 2.54#	15.50 ± 3.95#
9,10-EpOME	57.35 ± 10.34	470.00 ± 119.10*	105.13 ± 32.13‡	181.15 ± 46.75‡	14.38 ± 2.70	9.18 ± 1.76	30.04 ± 7.38	25.94 ± 8.75
14,15-EET	10.88 ± 1.24	16.19 ± 2.91	9.59 ± 2.68	13.09 ± 4.18	1.64 ± 0.05	1.33 ± 0.10	3.65 ± 0.45#	2.53 ± 0.67
11,12-EET	6.74 ± 0.86	16.88 ± 2.94*	6.02 ± 1.56‡	8.38 ± 2.77	1.34 ± 0.19	0.94 ± 0.15	2.60 ± 0.40#	2.04 ± 0.58
8,9-EET	12.63 ± 1.88	31.93 ± 5.77*	12.25 ± 2.45‡	18.02 ± 3.54	1.45 ± 0.28	2.04 ± 0.71	3.69 ± 0.94	3.63 ± 1.06
5,6-EET	ND	ND	ND	ND	ND	ND	ND	ND
17,18-EpETE	ND	ND	ND	ND	0.65 ± 0.11	0.54 ± 0.19	2.60 ± 0.47#	2.98 ± 0.75#
19,20-EpDPE	35.63 ± 2.32	22.54 ± 4.93	15.24 ± 2.54*	14.78 ± 4.98*	47.66 ± 6.25	39.40 ± 9.46	69.20 ± 8.48	44.89 ± 12.10
Soluble epoxide hydrolase-dependent metabolism								
12,13-diHOME	6.09 ± 1.60	14.00 ± 2.32*	7.25 ± 1.81	10.93 ± 1.64	2.08 ± 0.18	2.22 ± 0.49	1.37 ± 0.05	1.51 ± 0.40
9,10-diHOME	11.98 ± 3.79	76.27 ± 25.19*	20.15 ± 5.02‡	32.88 ± 6.40	2.44 ± 0.41	2.50 ± 0.68	3.62 ± 0.70	4.95 ± 2.07
14,15-DHET	0.48 ± 0.07	0.62 ± 0.06	0.47 ± 0.07	0.62 ± 0.07	0.17 ± 0.01	0.27 ± 0.08	0.20 ± 0.02	0.16 ± 0.04
11,12-DHET	0.85 ± 0.12	0.96 ± 0.19	0.54 ± 0.09	0.70 ± 0.11	0.35 ± 0.05	0.40 ± 0.12	0.38 ± 0.04	0.42 ± 0.07
8,9-DHET	0.68 ± 0.12	2.02 ± 0.17*	0.88 ± 0.18‡	1.72 ± 0.69	0.36 ± 0.04	0.53 ± 0.15	0.53 ± 0.15	0.57 ± 0.12
5,6-DHET	0.45 ± 0.15	0.92 ± 0.16	0.53 ± 0.10	0.41 ± 0.09	0.26 ± 0.02	0.39 ± 0.10	0.37 ± 0.03	0.32 ± 0.10
Epoxygenase:Diol Metabolite Ratios								
12,13-EpOME:DiHome	5.10 ± 1.42	10.27 ± 1.21*	3.22 ± 0.29#	4.53 ± 1.05#	2.43 ± 0.22	2.68 ± 0.68	7.52 ± 0.53#	10.21 ± 0.58#
9,10-EpOME:DiHome	7.40 ± 0.70	6.64 ± 0.57	5.23 ± 0.96	3.82 ± 0.70*	6.04 ± 0.77	6.32 ± 1.38	8.50 ± 1.35	5.61 ± 0.56
14,15-EET:DHET	23.54 ± 3.13	25.57 ± 3.7	19.72 ± 5.49	23.43 ± 7.43	9.68 ± 0.70	9.25 ± 2.18	18.70 ± 2.64#	16.18 ± 3.20
11,12-EET:DHET	8.26 ± 1.03	19.93 ± 4.51	11.34 ± 2.89	11.65 ± 3.26	3.85 ± 0.23	4.23 ± 0.92	5.92 ± 1.11	4.67 ± 0.61
8,9-EET:DHET	20.43 ± 3.61	15.63 ± 2.11*	14.58 ± 3.21	16.84 ± 11.22	4.85 ± 0.20	6.55 ± 1.79	6.74 ± 1.33	6.12 ± 0.74
ω-Hydrolase-dependent metabolism								
19-HETE	ND	ND	ND	ND	0.79 ± 0.08	1.18 ± 0.17	0.89 ± 0.18	0.37 ± 0.09
20-HETE	ND	ND	ND	ND	0.51 ± 0.07	0.66 ± 0.06	1.27 ± 0.30#	0.65 ± 0.03
Lipoxygenase-dependent metabolism								
13-HODE	542.15 ± 204.56	1468.50 ± 308.42*	297.93 ± 97.06‡	413.72 ± 166.53‡	54.52 ± 11.64	40.46 ± 14.01	55.94 ± 4.41	58.38 ± 19.74
9-HODE	359.43 ± 154.53	1014.17 ± 204.83*	217.27 ± 64.37‡	283.35 ± 116.04‡	31.39 ± 3.76	29.88± 11.34	40.91 ± 3.94	43.56 ± 15.81
15-HETE	48.83 ± 7.97	30.37 ± 4.22	25.67 ± 6.85	20.32 ± 7.94*	6.83 ± 0.64	9.67 ± 3.88	11.78 ± 1.10	11.76 ± 3.25
11-HETE	33.79 ± 5.77	28.03 ± 5.73	17.29 ± 3.40	13.57 ± 5.09	2.54 ± 0.22	2.94 ± 0.84	3.71 ± 0.05	4.15 ± 0.87
12-HETE	45.64 ± 7.85	23.51 ± 4.94	19.72 ± 5.12*	13.47 ± 5.29*	7.91 ± 1.45	7.84 ± 3.10	5.95 ± 1.04	7.35 ± 1.98
8-HETE	497.33 ± 97.29	321.02 ± 67.93	312.65 ± 71.92	179.97 ± 70.71*	ND	ND	ND	ND
5-HETE	35.98 ± 5.83	25.35 ± 4.94	19.10 ± 3.24	12.52 ± 3.85*	1.60 ± 0.28	1.51 ± 0.51	1.28 ± 0.22	1.54 ± 0.41

Cyclooxygenase-dependent metabolism								
6ketoPGF$_{1\alpha}$	5.70 ± 1.07	0.60 ± 0.09	3.76 ± 2.50	3.32 ± 2.29	3.41 ± 0.25	7.28 ± 2.34	4.86 ± 0.72	9.08 ± 1.84
TXB$_2$	0.23 ± 0.06	0.11 ± 0.06	0.07 ± 0.04	0.07 ± 0.04	0.48 ± 0.07	1.06 ± 0.31	0.66 ± 0.08	1.03 ± 0.19
PGF$_{2\alpha}$	1.12 ± 0.49	1.09 ± 0.31	3.22 ± 0.81	1.29 ± 0.61	ND	ND	ND	ND
PGE$_2$	8.99 ± 2.66	25.99 ± 3.63*	9.59 ± 2.93‡	12.73 ± 2.76‡	ND	ND	ND	ND
8isoPGF$_{2\alpha}$	1.06 ± 0.09	1.52 ± 0.20	0.94 ± 0.33	0.95 ± 0.05	0.15 ± 0.02	0.50 ± 0.26	0.55 ± 0.11	0.45 ± 0.15
PGB$_2$	ND	ND	ND	ND	ND	ND	ND	ND
PGD$_2$	16.52 ± 4.38	47.35 ± 6.82	33.00 ± 18.19	82.77 ± 61.36	0.47 ± 0.05	0.53 ± 0.22	0.62 ± 0.13	1.05 ± 0.32

Supplemental Table 3. Cardiac functional parameters in sham female mice at baseline and following treatment with *t*AUCB for 7 and 28 days measured by 2D echocardiography and electrocardiogram. Data are shown as mean ± SEM, N = 5-17 for ECHO. N = 1 for ECG due to low animal numbers. $P \leq 0.05$.

	WT+*t*AUCB-Sham		
ECHOCARDIOGRAPHY	Baseline	7d	28d
HR, beats/min	469 ± 18	474 ± 18	447 ± 13
Wall measurements			
Corrected LV mass, mg	90.3 ± 3.4	78.0 ± 6.1	116.7 ± 13.7
IVS-diastole, mm	0.78 ±0.05	0.76 ± 0.04	0.89 ± 0.05
IVS-systole, mm	1.16 ± 0.07	1.07 ± 0.01	1.31 ± 0.09
LVPW-diastole, mm	0.84 ± 0.03	0.79 ± 0.04	0.91 ± 0.02
LVPW-systole, mm	1.13 ± 0.02	1.16 ± 0.05	1.30 ± 0.06
LVID-diastole, mm	3.88 ± 0.16	3.66 ± 0.21	4.11 ± 0.22
LVID-systole, mm	2.61 ± 0.13	2.37 ± 0.17	2.67 ± 0.18
Cardiac Function, Simpsons			
EF, %	61.39 ± 1.06	62.11 ± 1.81	61.48 ± 0.35
FAC, %	52.76 ± 3.02	54.46 ± 1.08	53.58 ± 1.71
LVEDV, μl	67.98 ± 5.88	77.93 ± 6.64	71.42 ± 7.80
LVESV, μl	26.40 ± 2.95	29.65 ± 3.11	27.47 ± 2.86
CO, ml/min	20.31 ± 1.44	23.64 ± 1.43	20.48 ± 2.16
SV, μl	41.58 ± 3.0	48.28 ± 4.05	43.96 ± 4.95
Doppler Imaging			
IVRT, ms	14.68 ± 1.00	12.63 ± 0.39	15.91 ± 1.24
IVCT, ms	14.48 ± 1.66	12.70 ± 1.67	14.03 ± 0.79
ET, ms	43.65 ± 2.99	46.73 ± 2.15	43.00 ± 1.62
Tei index	0.67 ± 0.02	0.55 ± 0.06	0.70 ± 0.05
E'	23.35 ± 1.92	24.90 ± 1.15	25.24 ± 2.75
E'/A'	1.00 ± 0.13	1.06 ± 0.07	1.28 ± 0.06
E/E'	25.05 ± 2.10	23.53 ± 0.98	26.23 ± 2.93
ELECTROCARDIOGRAM			
HR, beats/min	502	573	513
RR, ms	119.5	104.7	116.9
QRS, ms	10	9.5	10.3
PR, ms	44.4	41.3	40.7
QTcF, ms	46.9	48.0	51.5

Supplemental Table 4. Cardiac functional parameters in female CYP2J2-Tr mice at baseline and 28 days post-MI measured by 2D echocardiography. Data are shown as mean ± SEM, N = 5-17. Data is from two-way ANOVA with Tukey's post-hoc test. *P* ≤ 0.05, *vs baseline; # vs WT counterpart.

	CYP2J2-Tr+*t*AUCB:0d	
	Baseline	28d
HR, beats/min	488 ± 11	481 ± 19
Wall measurements		
Corrected LV mass, mg	101.02 ± 4.55	177.45 ± 14.90*
IVS-diastole, mm	0.84 ± 0.02	0.91 ± 0.05#
IVS-systole, mm	1.21 ± 0.05	1.13 ± 0.08#
LVPW-diastole, mm	0.84 ± 0.03	0.94 ± 0.04
LVPW-systole, mm	1.26 ± 0.07	1.26 ± 0.10
LVID-diastole, mm	4.01 ± 0.08	5.18 ± 0.22*
LVID-systole, mm	2.49 ± 0.12	4.35 ± 0.28*
Cardiac Function, Simpsons		
EF, %	68.85 ± 2.02	31.85 ± 5.09*
FAC, %	62.78 ± 3.51	27.73 ± 5.67*
LVEDV, µl	63.59 ± 4.73	106.21 ± 11.96#
LVESV, µl	19.95 ± 2.21	73.96 ± 12.55*#
CO, ml/min	21.00 ± 1.46	15.37 ± 2.02
SV, µl	43.64 ± 3.01	32.35 ± 4.77
Doppler Imaging		
IVRT, ms	18.42 ± 0.89	21.47 ± 0.97
IVCT, ms	15.16 ± 1.87	14.35 ± 1.71#
ET, ms	43.32 ± 1.36	41.28 ± 2.54
Tei index	0.77 ± 0.06	1.01 ± 0.16
E'	26.92 ± 1.98	25.27 ± 8.91
E'/A'	1.10 ± 0.09	0.81 ± 0.10
E/E'	18.34 ± 1.68	33.40 ± 14.98

Supplemental Table 5. Cardiac functional parameters in female WT+tAUCB:0d treated mice at baseline and 28 days post-MI measured by 2D echocardiography. Data are shown as mean ± SEM, N = 5-10. Data is from two-way ANOVA with Tukey's post-hoc test. *P* ≤ 0.05; * vs Control, # vs WT group, ‡ vs non-infarct group.

	WT+*t*AUCB:0d Tx		
ECHOCARDIOGRAPHY	Baseline	7d	28d
HR, beats/min	435 ± 8	452 ± 8	502 ± 18
Wall measurements			
Corrected LV mass, mg	93.8 ± 7.8	138.9 ± 11.4	149.6 ± 18.8*
IVS-diastole, mm	0.81 ± 0.05	0.80 ± 0.05	0.72 ± 0.10
IVS-systole, mm	1.13 ± 0.07	1.11 ± 0.16	0.97 ± 0.13
LVPW-diastole, mm	0.82 ± 0.03	0.87 ± 0.11	0.78 ± 0.09
LVPW-systole, mm	1.15 ± 0.03	1.06 ± 0.18	0.94 ± 0.13
LVID-diastole, mm	3.92 ± 0.11	4.91 ± 0.36*	5.59 ± 0.31*
LVID-systole, mm	2.64 ± 0.11	4.13 ± 0.58*	5.01 ± 0.43*
Cardiac Function, Simpsons			
EF, %	61.14 ± 2.04	31.60 ± 7.55*	26.42 ± 5.37*
FAC, %	53.19 ± 2.86	17.96 ± 5.79*	20.30 ± 4.44*
LVEDV, µl	67.15 ± 5.86	168.10 ± 31.40*	170.04 ± 17.36*
LVESV, µl	26.34 ± 3.21	121.41 ± 30.05*	133.01 ± 19.43*
CO, ml/min	18.54 ± 1.44	22.38 ± 2.80	18.83 ± 1.65
SV, µl	40.81 ± 3.14	46.68 ± 9.21	37.02 ± 3.60
Doppler Imaging			
IVRT, ms	15.84 ± 1.37	16.17 ± 2.96	23.13 ± 6.79
IVCT, ms	17.56 ± 2.80	29.72 ± 10.63	17.42 ± 3.45
ET, ms	44.34 ± 1.97	40.47 ± 2.24	39.72 ± 4.36
Tei index	0.76 ± 0.08	1.24 ± 0.47	1.12 ± 0.52*#‡
E'	21.03 ± 1.83	22.34 ± 6.82	18.76 ± 2.76
E'/A'	1.19 ± 0.05	1.18 ± 0.06	1.15 ± 0.12
E/E'	25.99 ± 2.29	20.31 ± 3.49	29.15 ± 3.41
ELECTROCARDIOGRAM			
HR, beats/min	489 ± 20	467 ± 17	494 ± 23
RR, ms	123.8 ± 4.8	129.1 ± 5.8	118.9 ± 6.5
QRS, ms	11.4 ± 0.3	11.3 ± 0.7	10.5 ± 1.8#
PR, ms	41.7 ± 0.7	42.0 ± 1.1	47.4 ± 1.3*
QTcF, ms	49.8 ± 3.4	71.5 ± 2.1*	55.2 ± 2.9‡

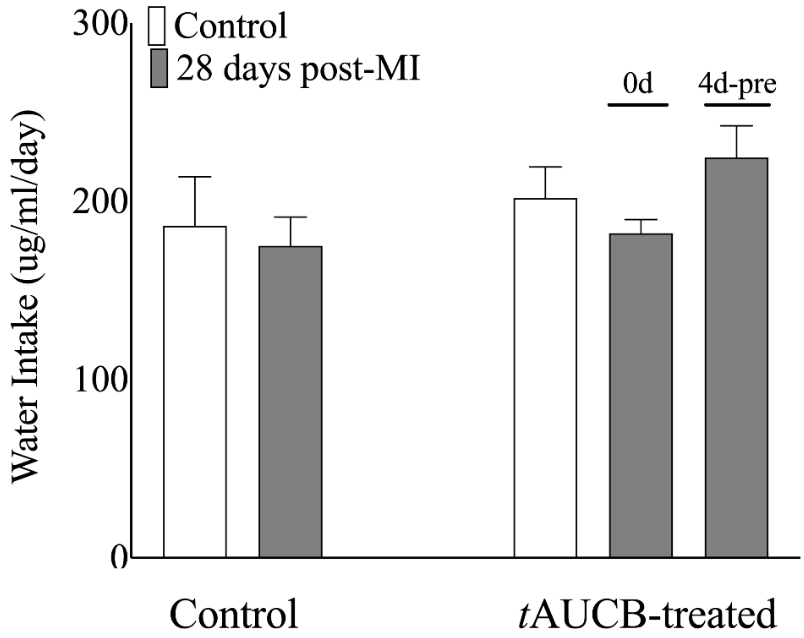

Supplemental Figure S1. Water intake in female WT and *t*AUCB treated mice over the 28 days did not differ between groups. Only animals that survived are represented. Data are represented as mean ± SEM. N = 3-12. $P \leq 0.05$.

Supplemental Figure 2.

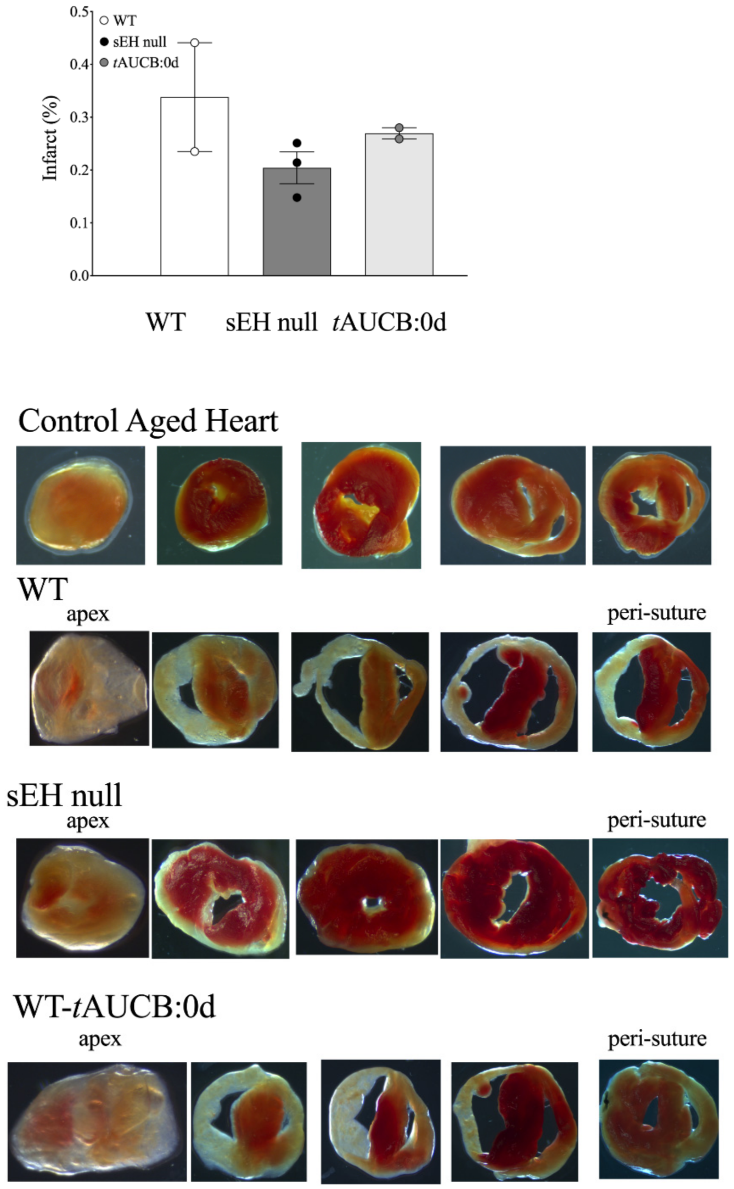

Supplemental Figure S2. Development of infarct measured by tetrazolium chloride (TTC) in WT, sEH null and *t*AUCB:0d in female mice. Data are represented as mean ± SEM. Representative images are below. All hearts were confirmed to have infarct at time of necropsy.

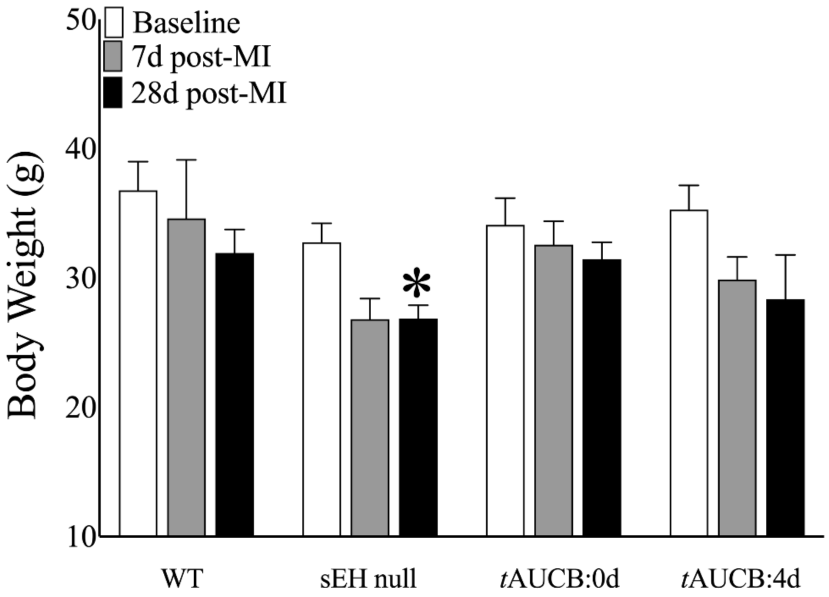

Supplemental Figure S3. Body weight measured at baseline, 7d and 28 days post-MI in female WT, sEH null and *t*AUCB treated mice. Data are represented as mean ± SEM. N = 5-22. $P \leq 0.05$; *vs baseline.

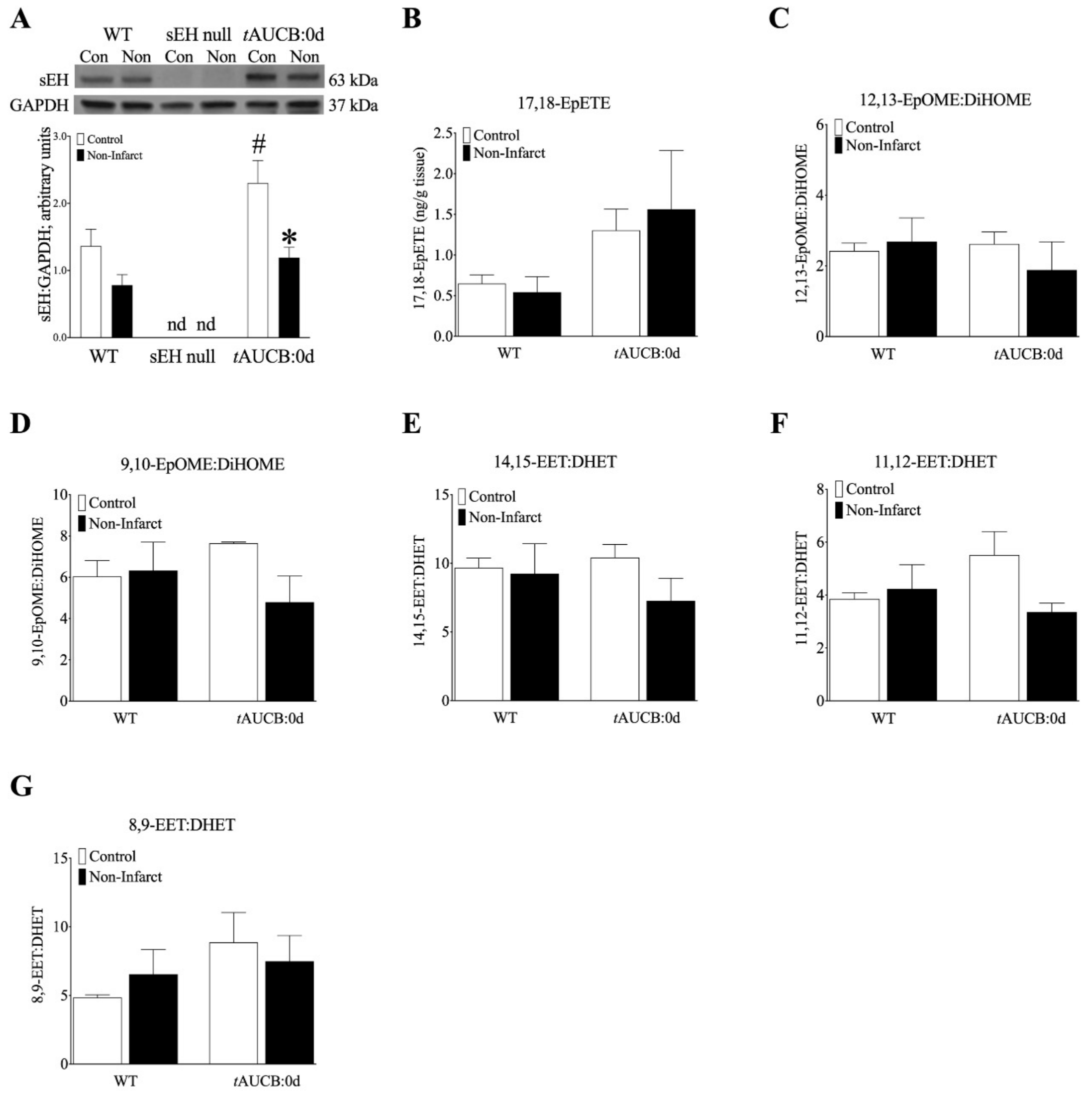

Supplemental Figure S4. Epoxide hydrolase expression and oxylipid metabolites (ng/g tissue) in female left ventricular tissue from mice with *t*AUCB:0d treatment. (A) Soluble epoxide hydrolase protein expression for female mice with same-day *t*AUCB treatment compared to WT. Only control and non-infarct are represented. Data are represented as mean ± SEM. (B-G) Oxylipid metabolite ratios in WT compared to WT+*t*AUCB:0d treatment. (B) 17,18-EpETE, (C) 12,13-EpOME:DiHOME ratios, (D) 9,10-EpOME:DiHOME ratios, (E) 14,15-EET:DHET ratios, (F) 11,12-EET:DHET ratios, (G) 8,9-EET:DHET ratios. Data are mean ± SEM, n = 3-6. In all cases, $P \leq 0.05$; *vs control. # vs WT group.

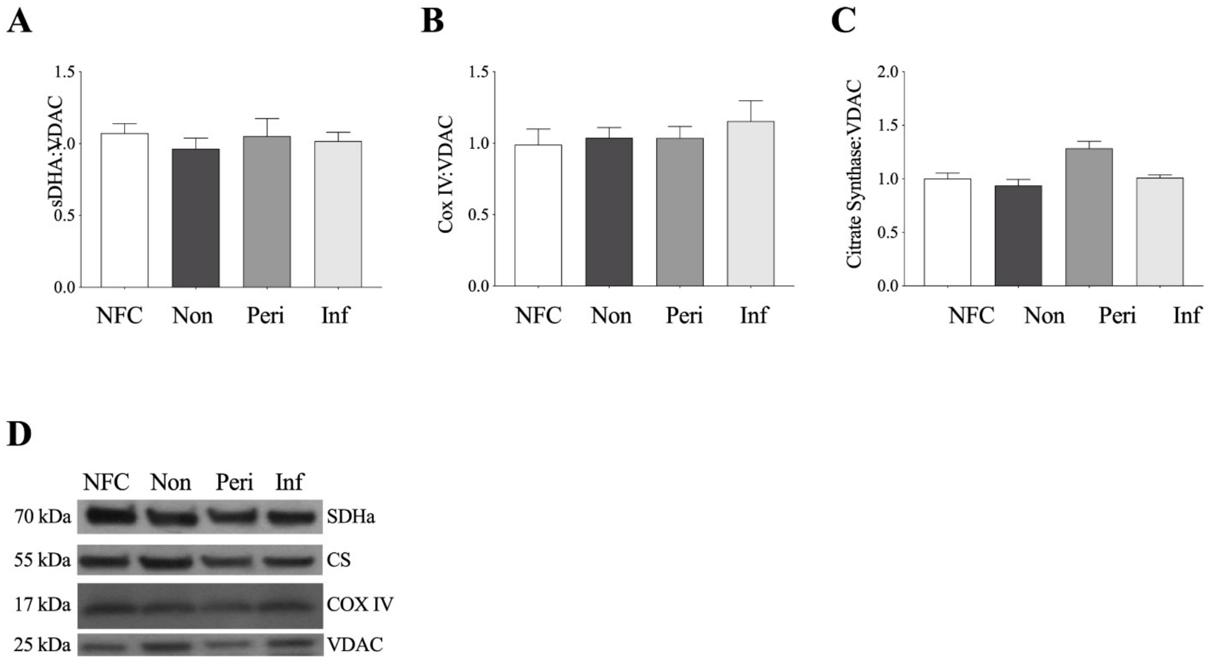

Supplemental Figure S5. Protein expression of key mitochondrial ETC enzymes in mitochondrial fractions from human female NFC and ICM hearts. (A) Succinate dehydrogenase A (SDHA) expression, (B) Cytochrome C oxidase (COX IV) expression, (C) Citrate synthase (CS) expression, (D) representative blots. All proteins were normalized to VDAC. Data are represented as mean ± SEM, N = 3-5. $P \leq 0.05$.

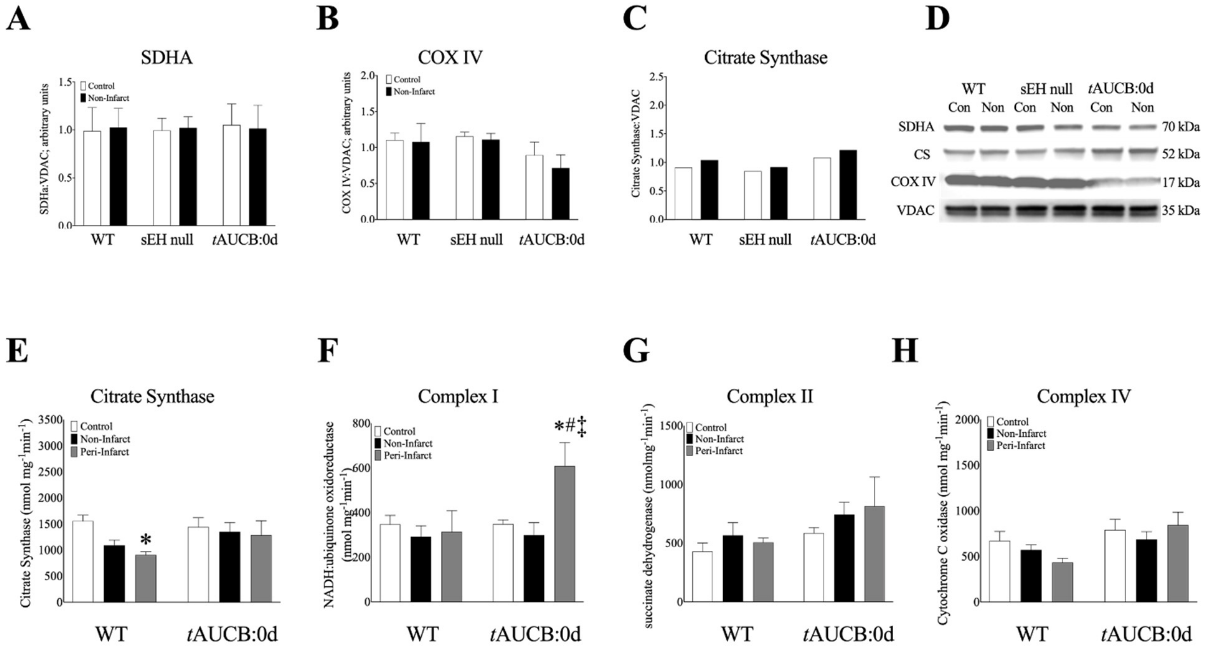

Supplemental Figure S6. Electron transport chain protein expression and activity in female mouse hearts with *t*AUCB:0d treatment. (A-D) Protein expression of key mitochondrial ETC enzymes in mitochondrial fractions from female mouse left ventricular tissue. (A) Succinate dehydrogenase A (SDHA) expression, (B) Cytochrome C oxidase (COX IV) expression, (C) Citrate synthase (CS) expression, (D) representative blots. All proteins were normalized to VDAC. (E-H) ETC complex activity (nmol $mg^{-1}min^{-1}$) in WT and *t*AUCB:0d treated female left ventricular tissue. (E) Citrate synthase (CS) activity, (F) Complex I (NADH:ubiquinone oxidoreductase) activity, (G) Complex II (succinate dehydrogenase, SDH), and (H) Complex IV (cytochrome C oxidase, COX IV). Data are represented as mean ± SEM, n = 3-9. $P \leq 0.05$; * vs Control, # vs WT group, ‡ vs non-infarct group.

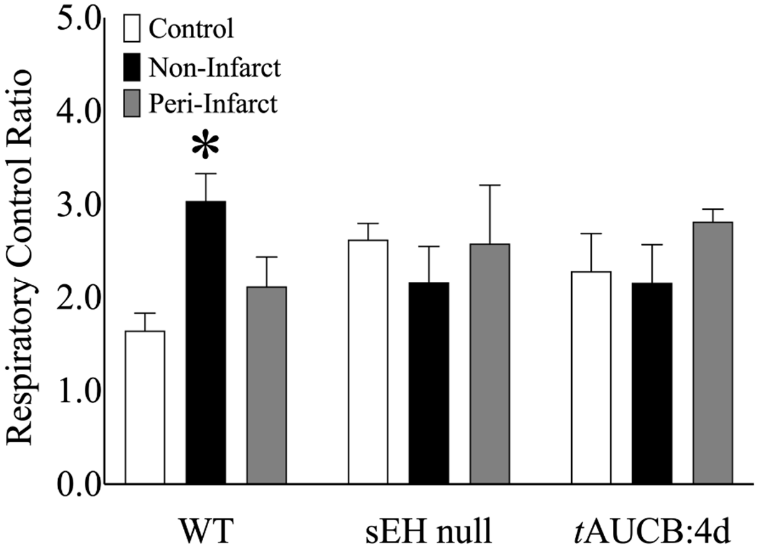

Supplemental Figure S7. Respiratory control ratio (RCR) in whole cardiac fibres taken from WT, sEH null and *t*AUCB-treated female mice. Data are means ± SEM, N = 3-6. $P \leq 0.05$; * vs Control.

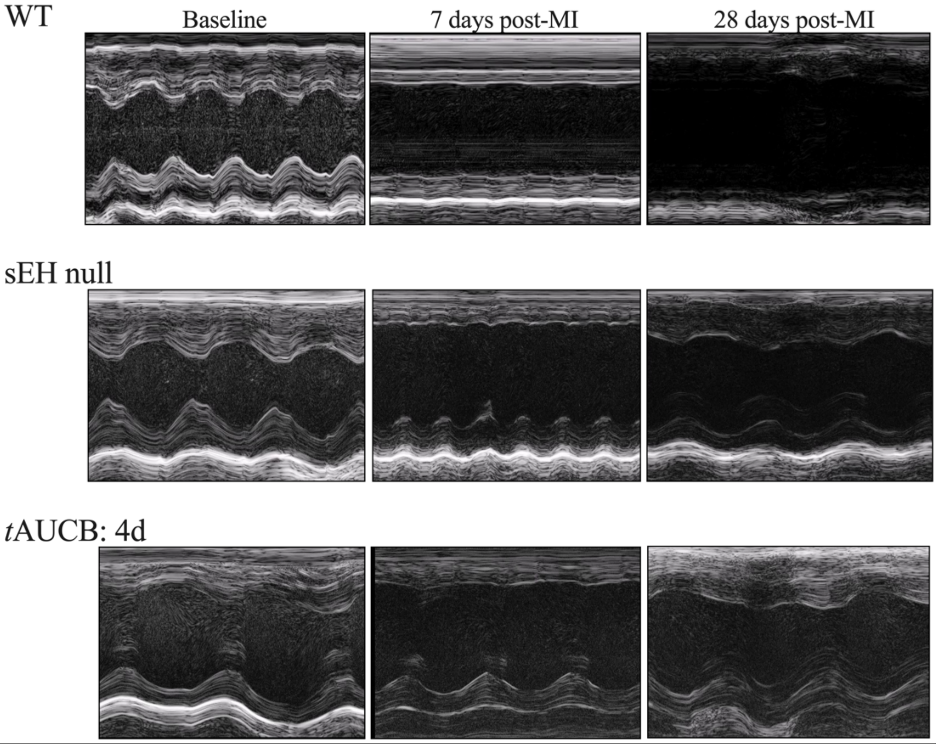

Supplemental Figure S8. Representative m-mode images from 2D echocardiography. M-mode slices were taken in SAX mode at level 2 (mid-papillary) at baseline, 7 and 28 days post-MI. M-mode images were used for quantitative wall measurements.
